# Knowledge and awareness of mitochondrial diseases among physicians in the tertiary hospitals in Ghana

**DOI:** 10.1371/journal.pone.0276549

**Published:** 2022-10-20

**Authors:** Eric A. Mensah, Bismark Sarfo, Alfred E. Yawson, Joshua Arthur, Augustine Ocloo

**Affiliations:** 1 Department of Biochemistry, Cell & Molecular Biology, School of Biological Sciences, College of Basic and Applied Sciences, University of Ghana, Legon, Accra, Ghana; 2 West African Centre for the Cell Biology of Infectious Pathogens, University of Ghana, Legon, Accra, Ghana; 3 Department of Epidemiology and Disease Control, School of Public Health, College of Health Sciences, University of Ghana, Legon, Accra, Ghana; 4 Department of Community Health, University of Ghana Medical School, College of Health Sciences, University of Ghana Korle Bu, Accra, Ghana; 5 Public Health Unit, Komfo Anokye Teaching Hospital, Kumasi, Ghana; Ospedali Riuniti di Foggia: Azienda Ospedaliera Universitaria Foggia, ITALY

## Abstract

**Background:**

Mitochondrial diseases/disorders (MDs), for decades, have been identified as a key underlying condition for many chronic diseases globally. However, data on the knowledge and prevalence of MDs in many countries in sub-Saharan Africa are lacking. This study assessed the knowledge, and awareness, of MDs among senior medical doctors in the five tertiary hospitals in Ghana.

**Method:**

Data were collected from one hundred and twenty-eight (128) medical doctors in the five Tertiary Hospitals in Ghana using both closed and open-ended questionnaires and analysed using descriptive statistics.

**Results:**

Of the 128 respondents, 70.32% were senior medical officers and above, 87% of them indicated that they were aware of MDs and over 90% said physicians do not often diagnose MDs in Ghana. About 81% indicated that MDs are associated with chronic illnesses whilst 72% said the disease is diagnosed in both males and females. About 45% of the respondents alluded to the fact that MDs are difficult to diagnose, are associated with mutations in both the mitochondrial and the nuclear DNA, and are non-infectious diseases. Approximately 85% said nervous system dysfunction and muscle weakness are some of the symptoms associated with MDs whilst 77% said fatigue is also one of the symptoms. About 38% of the respondents specified that they encounter myopathies. A majority (70%) did not know about the availability of any consensus or standard diagnostic procedure and/or drugs for MDs.

**Conclusion:**

There is a high level of knowledge and awareness of MDs among the respondents. However, there is a low disease encounter, which could be due to a lack of diagnostic protocols or a low disease prevalence. It is, therefore recommend that a patient perspective study, which looks at clinical records and laboratory data be conducted to fully ascertain the prevalence of MDs in Ghana and that appropriate educational strategies and interventions aimed at improving the diagnosis of mitochondrial diseases in Ghana be put in place.

## 1. Introduction

Mitochondrial diseases/disorders (MDs) constitute a heterogeneous group of non-infectious disorders characterized by impaired energy production caused by genetically based oxidative phosphorylation malfunction [1, 2]. They are a group of chronic diseases that are now known to be more common than initially thought, with increasing prevalence across the world, although they were once considered rare and obscure diseases affecting about 1 or 2 people per a million of the population [2]. Many MDs are so new that they have not yet been mentioned in medical textbooks or medical literature. Due to unpredictable epigenetic and genetic and genotypic and phenotypic mito-nuclear relationships, these diseases are collectively named “mystondria” (mysterious diseases of mitochondria) [3]. They can affect any organ in the body at any age and are severely debilitating, often fatal, and characteristically complex in nature [4, 5].

In recent times, the spectrum of MDs has increased, and are no rarer diseases but a group of disorders that can affect 1 in 200–250 people with clinical features varying between children and adults, and severity could range from asymptomatic mutation carriers to life-threatening illnesses [1]. MDs affect any and multiple tissues and organs of the human body, but in most cases, organs of higher energy demand such as the brain, heart, and skeletal muscle are greatly affected [4, 6]. These genetically oriented diseases, in many cases, are caused by mitochondrial DNA (mtDNA) mutations, with some contributions from mutations in nuclear DNA that may directly or indirectly interfere with mitochondrial oxidative phosphorylation [5–8]. Mitochondrial DNAs are highly susceptible to mutations and are therefore heterogeneous with the African populations harbouring the highest diversity [9, 10]. The presence of the slightest mtDNA sequence variations or single nucleotide polymorphisms confers risk for diseases [6]. Point mutations in mitochondrial transfer RNA (tRNA) and protein-encoding genes are the most common primary mutations associated with several MDs [11]. These primary mtDNA defects form the basis for a wide spectrum of human neurological and non-neurological diseases such as myopathy, peripheral neuropathy, seizures, dementia, stroke-like episodes, deafness, diabetes, kidney and liver dysfunctions, cardiomyopathy, endocrinopathies, skin disorders, and haematological dysfunctions [10, 12].

There have been suggestions that mitochondrial disorders should be routinely considered in chronic, progressive, and rare disease conditions [2, 3]. Unfortunately, a proper and straightforward diagnosis of MDs is currently lacking due to the lack of specific and reliable biomarkers [13, 14]. Even though diagnostic methods for MDs are available, there are limitations in the diagnostic approach, including overlapping phenotypes, patient selection, disease monitoring, and response to treatment, to name but a few [2, 7, 15, 16]. The current gold standard for the diagnosis of MDs is measuring the activity of the respiratory chain enzymes in tissue biopsies plus complex V and functional tests if fresh samples are available, in combination with other assessments. These assessments include brain imaging, genetic testing for specific mutations, histochemical investigations as well as exercise stress tests to determine the arteriovenous oxygen difference (a-vO_2_ difference) [17–19]. Over the past 15 years, various scoring systems for MDs have been developed (for both pediatric and adult patients) to assist physicians in screening patients for the disease [2, 20–24]. Nevertheless, the absence of standard diagnostic procedures makes it difficult for clinicians to diagnose patients with MDs all over the world [25] resulting in many mitochondrial disease conditions being never diagnosed and treated especially in resource-constrained countries [26].

Consequently, there is a paucity of data on MDs in Sub-Saharan Africa. Thus apart from very few publications mainly from South Africa on paediatric patients [7, 27, 28], there is a lack of data on the prevalence and incidence of MDs in many Sub-Saharan African countries. In addition, data on knowledge and awareness of MDs are lacking. The present study, therefore, assessed the knowledge, and awareness, of MDs among senior medical doctors in the five tertiary hospitals in Ghana. Medical doctors from these hospitals were assessed for their awareness, knowledge, and perception of the prevalence of mitochondrial diseases among patients in Ghana.

## 2. Materials and methods

### 2.1 Ethical consideration

The study was approved by the Ethics Committee of the College of Basic and Applied Sciences, University of Ghana (ECBAS 012/20-21), Institutional Review Board of the Korle Bu Teaching Hospital (KBTH-STC/IRB/00065/2021), the Ethical Review Committee of the Cape Coast Teaching Hospital (CCTHERC/EC/2021/050) and the Institutional Review Board of the Komfo Anokye Teaching Hospital (KATH IRB/CA/132/21). The ethical review certificates were shown to the participants after which verbal consent was sought for their participation in the study.

### 2.2 Study sites

The study sites were the Departments of Medicine or related departments that specialize in chronic non-communicable diseases in the five tertiary hospitals in Ghana (Korle-Bu Teaching Hospital (KBTH), Okomfo Anokye Teaching Hospital (KATH), Cape Coast Teaching Hospital (CCTH), Ho Teaching Hospital (HTH) and Tamale Teaching Hospital (TTH)). These five hospitals were chosen because of their function as referral hospitals.

The KBTH is the biggest referral centre in Ghana and the third biggest referral centre in Africa. It is located in the Capital city (Accra) of Ghana. It is the Teaching Hospital for the University of Ghana. It has 2,000 beds, 21 clinical and diagnostic departments, and three Centres of Excellence. It also has an average outpatient attendance of 1,500 with about 250 inpatient admissions daily.

The KATH is in the capital city (Kumasi) of the Ashanti region. It is the Teaching Hospital for the Kwame Nkrumah University of Science and Technology. It is the second-largest hospital in Ghana and the only tertiary health institution in the middle belt of the country. It is the main referral hospital for the Ashanti, Brong Ahafo (BA), Northern, Upper East, and Upper West Regions. Statistical records from KATH show that about two-thirds of the patients are from the Ashanti region, with BA and the three Northern regions sharing the remaining in a two-to-one ratio.

The CCTH is located in the capital city (Cape Coast) of the central region of Ghana. It is the teaching Hospital of the University of Cape Coast. The 400-bed hospital serves as the main referral facility in the central and western parts of the country.

The HTH is located in the capital city (Ho) of the Volta. It is the Teaching Hospital for the University of Health and Allied Sciences, Ghana. It is a tertiary care facility with a staff strength of about 1200, a bed capacity of 306 and 14 wards, and the main referral facility providing health services to the people in both the Volta region and the Oti region.

The TTH is located in the eastern part of the Tamale Metropolis in the capital city (Tamale) of the Northern region. It is the Teaching Hospital for the University of Development Studies. It serves as a major referral center for Northern Ghana. The Hospital also receives clients from some parts of Burkina Faso and Togo. The Hospital currently has a bed capacity of four hundred and eighty-four (484) beds. The Hospital provides its services to over a hundred thousand patients every year.

### 2.3 Study population and sample size

The target study population was professional medical practitioners consisting of consultants, specialists, and medical officers. The sample size was calculated using the sample size determination by Miller and Brewer [29]:

$$n=N/[1+N(\alpha^{2})]$$

wheren=samplesize,N=targetpopulationandα=errorterm.

The sample size was calculated using an estimated target population of 284, consisting of consultants, doctors in residence, specialists, and medical officers in the five selected hospitals and with an error term of 0.05.


$$n=284/[1+284(0.05^{2})]$$

n=166

Convenience proportionate sampling was used to collect the data from participants at each of the study sites (Table 1) using the formula:

$$n_{s}=(N_{s}/N)\;x\;n$$

where n_s_ = sample size at each site and N_s_ = target population at each site.

**Table 1:** Estimated sample size per site.

Site	Target population (N_s_)	Sample size (n_s_)
Cape Coast	12	7
Ho	12	7
Korle-Bu	168	98
Komfo Anokye	80	47
Tamale	12	7
**Total**	**284**	**166**

### 2.4 Data collection

The data were collected through the use of questionnaires that had been developed for the study. To avoid putting undue pressure on the medical doctors due to their busy schedules, the researchers identified staff at the facility who acted as contact persons. All the questionnaires were handed over to the contact person, who subsequently handed them out to the medical practitioners and also retrieved the completed questionnaires.

### 2.5 Inclusion and exclusion criteria

The present study included medical doctors, who specialized in handling chronic non-communicable diseases. Medical doctors who are not in active service and not specialized in managing chronic non-communicable diseases and house officers were excluded.

### 2.6 Analysis of data

The data were analyzed using descriptive statistics, such as proportions and percentages using STATA Version 15.

## 3. Results

### 3.1 Demographic characteristics of participants

Table 2 shows the demographic characteristics of the respondents. A total of 128 Physicians were surveyed, which were made up of 9 consultants (senior specialists physicians involved in the training of medical students), 21 specialists (specialist physicians involved in the training of medical students), 60 senior medical officers (senior general physicians working in the internal medicine or related department in the teaching hospital), and 38 medical officers (general physicians working in the internal medicine or related department in the teaching hospital). Thus majority (90/128; 70.32%) of respondents are senior medical officers and above.

**Table 2:** Demographics of participant physicians.

	N/128	%
**Gender**		
Male	76	59.38
Female	52	40.62
**Years of medical practice**		
1–5	43	33.59
6–10	44	34.38
11–15	19	14.84
16–20	11	8.59
>20	11	8.59
**Rank**		
Medical Officer	38	29.69
Senior Medical Officer	60	46.88
Specialist	21	16.41
Consultant	9	7.03

### 3.2 Awareness of MDs among the study participants

Table 3 summarizes the extent of awareness of MDs among the respondents. The majority (111/128; 87%) of the respondents indicated that they are aware of MDs indicating a high level of awareness among them. Over 90% (119/128) indicated that physicians do not often diagnose MDs in Ghana. About 81% (104/128) indicated that MDs are associated with chronic illnesses whilst 72% indicated that the disease is diagnosed in both males and females. However, a small proportion indicated that they were not aware of MDs (13%). In addition, 17% indicated that they did not know whether MDs are associated with chronic illnesses or not.

**Table 3:** Awareness of MDs among physicians.

Questions	Responses	N/128	%
Are you aware of MDs?	Yes	111	86.72
	No	17	13.28
Are MDs common among Ghanaians?	Yes	18	14.06
	No	100	78.13
	Do not know	10	7.81
How often do Physicians diagnose patients with MD?	Not at all	29	22.66
	Not very often	90	70.31
	Often	6	4.69
	Very Often	0	0.00
	Do not know	3	2.34
Are MDs associated with chronic illness?	Yes	104	81.25
	No	2	1.56
	Do not know	22	17.19
Which gender is usually diagnosed with MDs?	Male	20	15.63
	Female	16	12.50
	Both male and female	92	71.88
What age categories are usually diagnosed with MDs?	<10	40	31.25
	10–20	26	20.31
	21–40	35	27.34
	41–60	17	13.28
	>60	10	7.81

### 3.3 Knowledge about the general description of and symptoms associated with MDs

**Fig 1:**
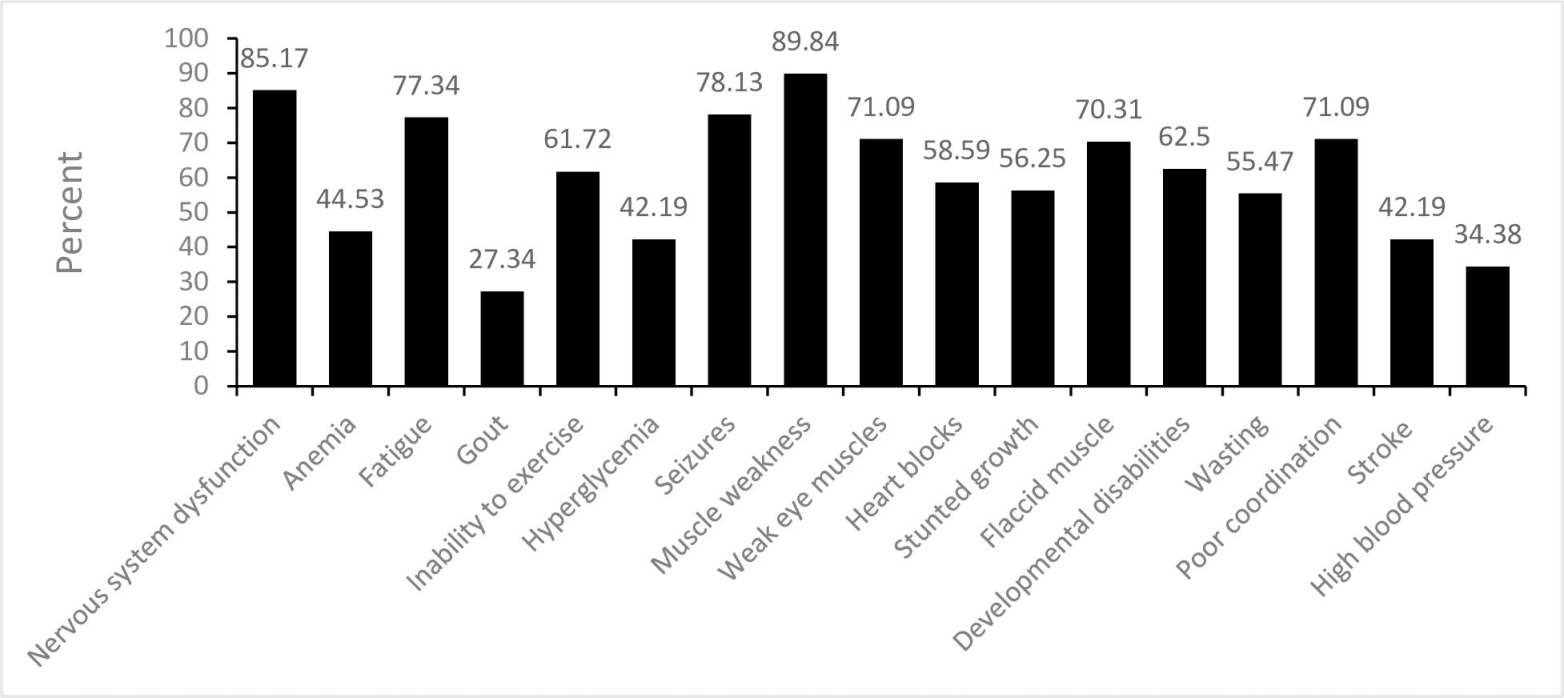
Knowledge of symptoms associated with mitochondrial diseases.

Table 4 summaries the knowledge of the respondents on the general description of MDs. Generally, the respondents demonstrated very good knowledge about the description of MDs. The majority alluded to the fact that MDs are difficult to diagnose (58/128; 45%), are associated with mutations in both the mitochondrial and the nuclear DNA (59/128; 46%), and are non-infectious diseases (44%). The respondents also demonstrated very good knowledge about the symptoms associated with MDs (Fig 1) as the majority specified that MDs are associated with nervous system dysfunction (85%), muscle weakness (90%), and fatigue (77%).

**Table 4:** General description of MDs.

Description	Responses	%
	(out of 128 participants)	
MDs are infectious diseases	4	3.23
MDs are non-infectious diseases	56	43.75
MDs are easy to diagnose	2	1.56
MDs are difficult to diagnose	58	45.31
MDs are genetic diseases associated with only the mitochondrial DNA	45	35.16
MDs are associated with both nuclear and the mitochondrial DNA	59	46.09

Participants were allowed to select multiple descriptions

### 3.4 Frequency of encounters with mitochondria-related disorders

**Fig 2:**
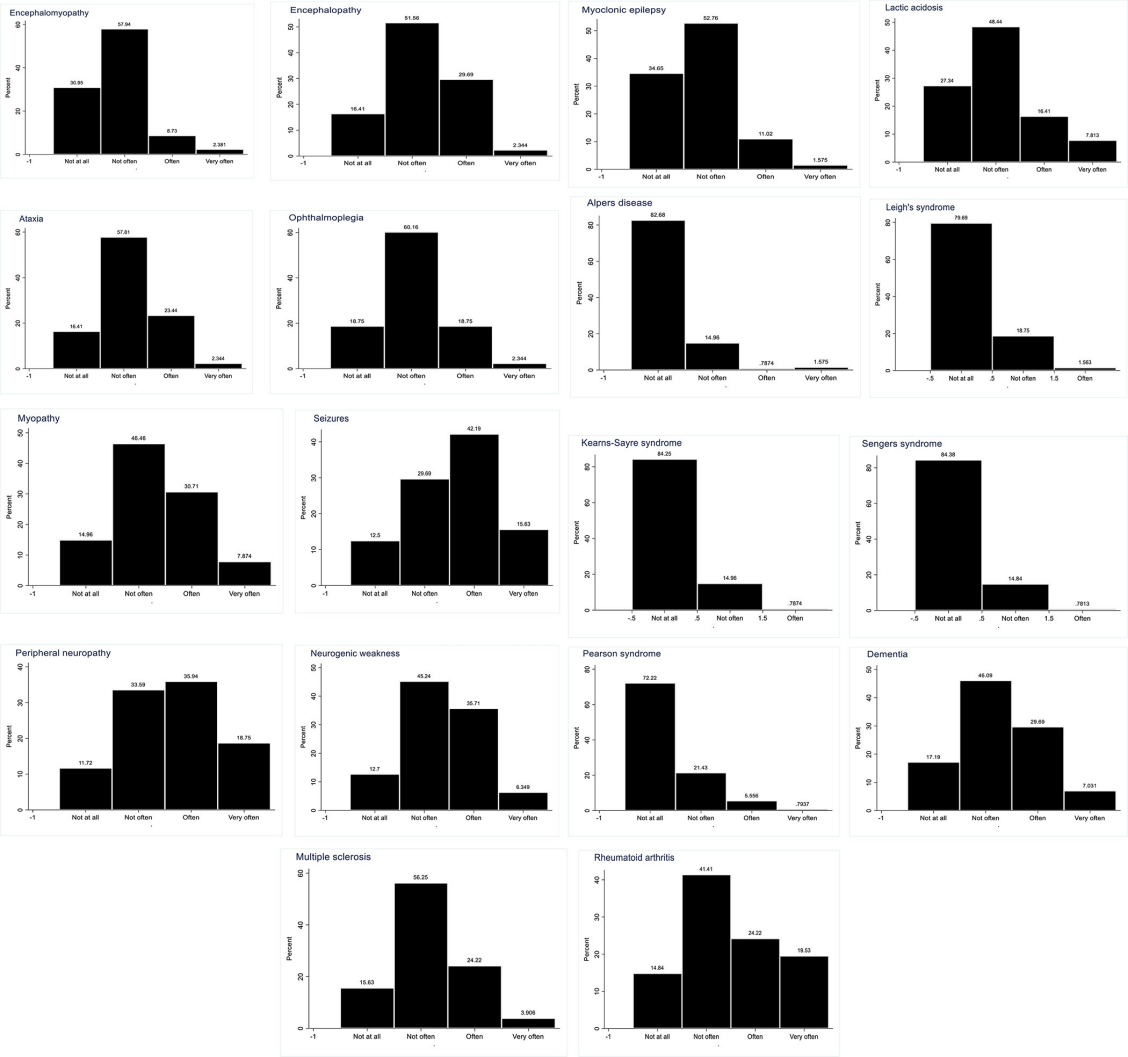
Frequency of encountering patients with specific mitochondrial-related disorders.

Fig 2 collated the frequency of encounters with mitochondria-related disorders by the respondents. Expectedly, seizures, peripheral neuropathy, and rheumatoid arthritis are the most common conditions encountered. Intriguingly, 38% of the respondents indicated that they encounter myopathies.

### 3.5 Availability of diagnostic protocols, approved drugs, and consensus treatment and/or management procedures for MDs

**Fig 3:**
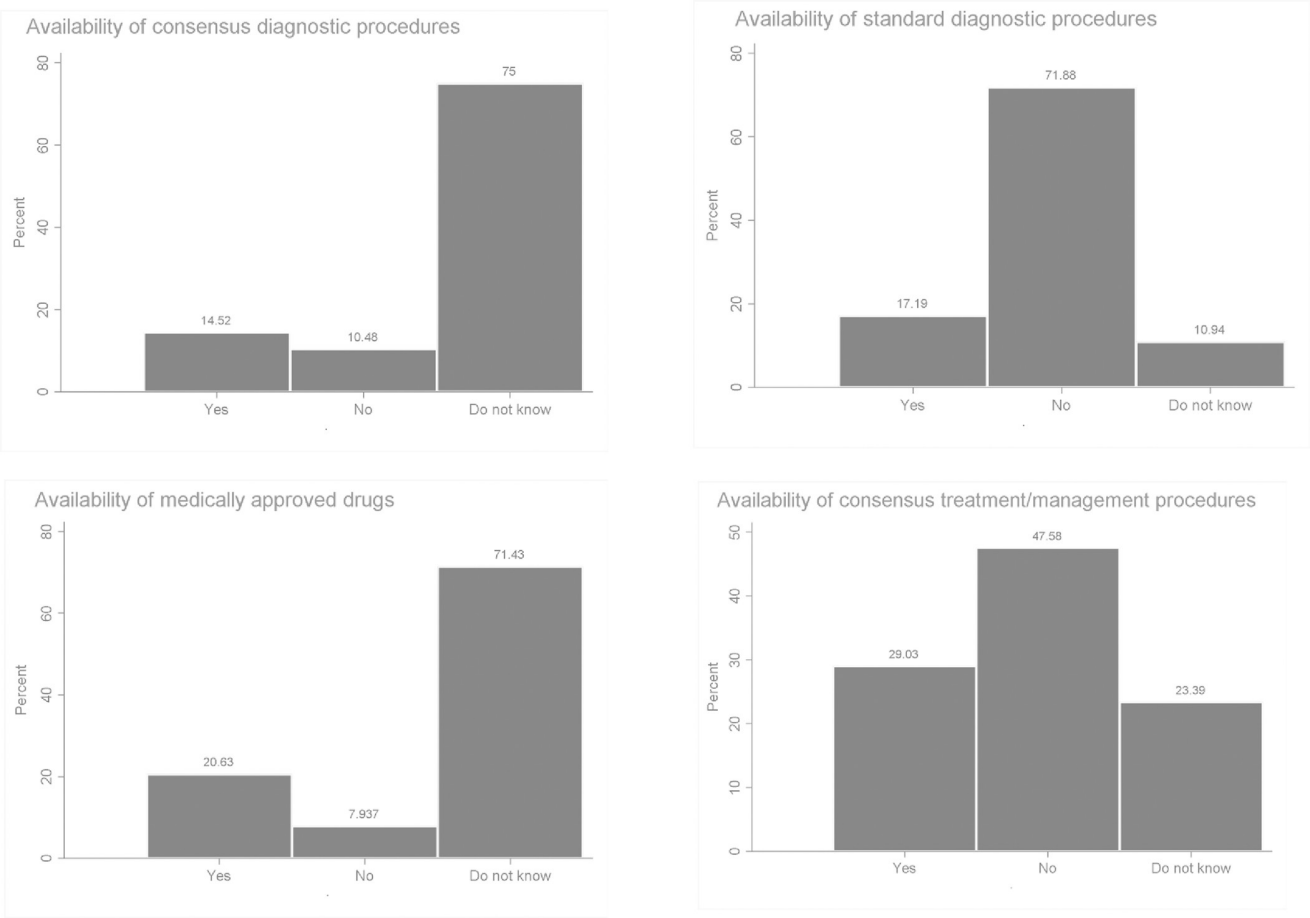
Mitochondrial disease diagnosis and treatment.

The responses of the participants to questions regarding the availability of MDs’ diagnostic protocols, approved drugs, and consensus treatment and/or management procedures are summarised in Fig 3. The majority (70%) of the respondents did not know about the availability of any consensus or standard diagnostic procedure, about 70% did not know about the availability of any drug for MDs and about 50% indicated that there were no consensus treatment/management procedures.

### 3.6 Patient education and level of understanding of MDs

**Fig 4:**
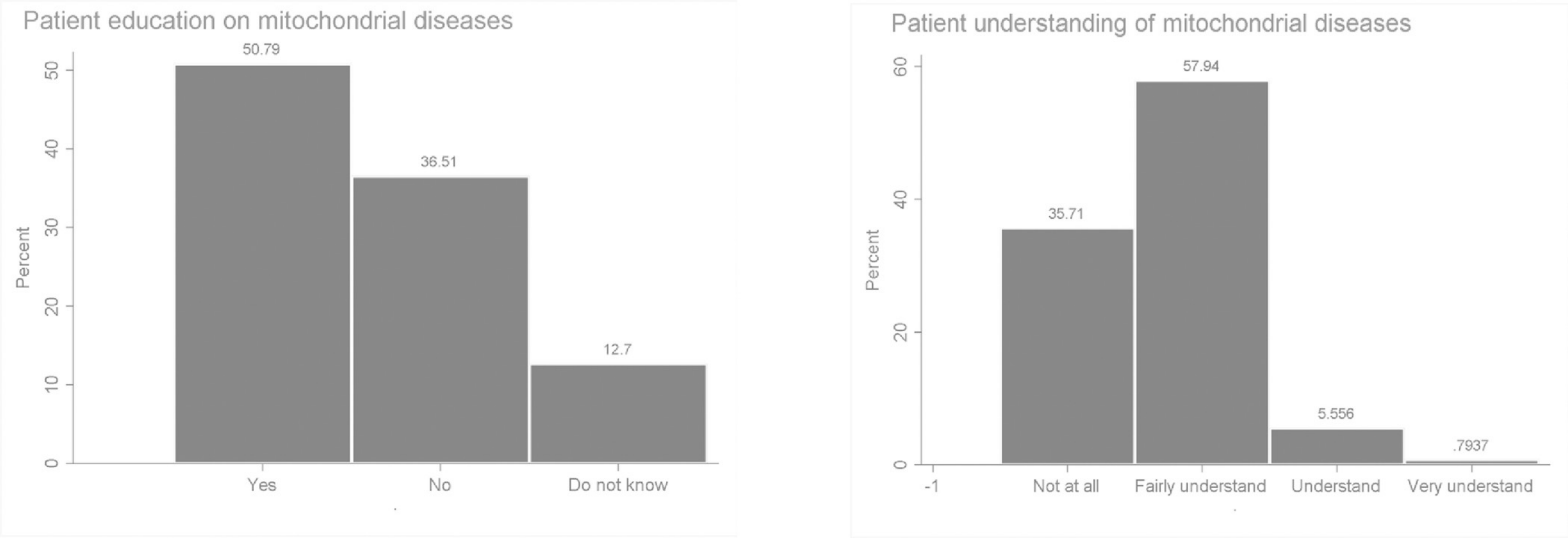
Patient education and level of understanding of mitochondrial diseases.

Fig 4 summarises the responses to questions regarding the education of patients by physicians about MDs and their level of understanding. About 50% of the respondents specified that patients are regularly educated on MDs and about 40% indicated that patients do not mostly understand the issues regarding mitochondrial diseases.

## 4. Discussion

The present study assessed the knowledge, and awareness, of MDs among senior medical doctors in the five tertiary hospitals in Ghana. To our knowledge, this is the first study that directly assessed knowledge and awareness of MD among physicians in sub-Saharan Africa. This study has become even more important because the COVID-19 pandemic has demonstrated a higher vulnerability of patients with chronic illnesses to opportunistic infections, resulting in a significant level of morbidity and mortality in that clinical evidence has shown that individuals with underlying disease conditions were more at risk of experiencing complications from COVID-19 infection [30]. Although there is no data on the extent of severity of COVID-19 complications among MD individuals, recent studies have shown possible dysfunction of mitochondrial oxidative function during COVID-19 pathogenesis [31], and for decades, mitochondrial dysfunction has been identified as a key underlying disease condition for many chronic diseases we know of today [1–3].

The majority of respondents in this study are senior medical officers and above. The respondents expressed a high level of awareness and knowledge about MDs as most of them alluded to the fact that they are aware of MDs and that MDs are associated with chronic illnesses and are diagnosed among both males and females. The respondents also demonstrated an impressive knowledge about the symptoms associated with MDs such that, most of them indicated that MDs are associated with nervous system dysfunction, muscle weakness, and fatigue among others. Most of the respondents thought MDs are not common among Ghanaians and almost all of them indicated that they do not diagnose MDs at all or not very often (Tables 3 & 4).

MDs are difficult to diagnose reliably, because of their wide clinical and genetic heterogeneity, and require a detailed medical history and extensive knowledge and expertise on behalf of the diagnosing physician [32]. Anecdotal evidence from clinicians, researchers, and patients suggests that before finally receiving a diagnosis of mitochondrial disease, many patients go through extensive clinical reviews, visit numerous clinical specialists, receive other conflicting diagnoses, and endure repeated and sometimes invasive testing [25, 33]. In addition, misdiagnoses and underdiagnoses are not uncommon [32]. This is especially true for resource-constrained settings as alluded to in a recent systematic review by Hettiarachchi and co-workers [26]. The exact prevalence and incidence of MDs in the world are also difficult to estimate. It is therefore not surprising that respondents noted that MDs are not common among Ghanaians and almost all of them indicated that they do not diagnose MDs at all or not very often. This is most likely because the facilities in Ghana lack the resources to diagnose MDs. Indeed, this is confirmed by the respondents in their responses to questions on the availability of MDs diagnostic protocols, where most indicated that there are no consensus diagnostic protocols or do not know about any. Conversely, the low encounter with MDs as indicated by the respondents could be a result of a low prevalence of MDs among Africans. Although MDs are now more common than initially thought affecting about 1 in 200–250 people [1] with a current minimum prevalence of one in every 5000 live births [33], data on incidence and prevalence in Africa is lacking.

MDs comprises a group of rare, about 1 in 4,300, debilitating genetic disorders [34]. The disorders feature a wide array of multisystemic manifestations that frequently include developmental abnormalities or regression, myopathy, seizures, dementia, hearing loss, blindness, strokes, diabetes mellitus, and premature death affecting both children and adults. Expectedly, in addition to seizures, peripheral neuropathy, and rheumatoid arthritis are the most common conditions encountered by the respondents in their practice in this study. This observation seems to suggest that the involvement of the mitochondria in some clinical cases in the various hospitals in Ghana cannot be ignored and needs to be thoroughly investigated.

## 5. Conclusion

In conclusion, the study revealed that the physicians in the selected teaching hospitals in Ghana are very much aware of MDs. They also demonstrated very good knowledge of the description of and symptoms associated with MDs. They are however of the view that MDs are not commonly diagnosed in Ghana and that there are no available MD diagnosis protocols. Thus suggesting that the low diagnosis is likely to be a result of a lack of diagnostic protocols or low disease prevalence. It is, therefore recommended that a patient perspective study, which looks at clinical records and laboratory data be conducted to fully ascertain the prevalence of MDs in Ghana. Additionally, we recommend that appropriate educational strategies and interventions aimed at improving the diagnosis of mitochondrial diseases in Ghana be put in place.

## 6. Limitations of this study

The limitations of the study include the fact that the participants were allowed to go home with the questionnaire and some of them could have referred to books or the internet before responding to the questionnaire. This will preclude genuine knowledge and awareness of MDs. Secondly, the participants were not asked to indicate their estimated percentage prevalence of MDs in Ghana. Thirdly, due to the busy nature of their schedule, verbal informed consent was obtained rather than written informed consent. Finally, the response rate of 77% (128 out of 166) is relatively low.

## Supporting information

S1 Data (XLSX)

## References

[1] Liang C, Ahmad K, Sue CM. The broadening spectrum of mitochondrial disease: shifts in the diagnostic paradigm. Biochimica et Biophysica Acta (BBA)-General Subjects. 2014 Apr 1;1840(4):1360–7. doi: 10.1016/j.bbagen.2013.10.040 24239706

[2] Schaefer AM, Taylor RW, Turnbull DM, Chinnery PF. The epidemiology of mitochondrial disorders—past, present and future. Biochimica et Biophysica Acta (BBA)-Bioenergetics. 2004 Dec 6;1659(2–3):115–20. doi: 10.1016/j.bbabio.2004.09.005 15576042

[3] Singh KK. MIPIGENETICS and MIPIGENOMICS: Integrating mitochondria-induced mayhem contributing to mystondria. Mitochondrion. 2015(24):S6.

[4] Smeitink JA. Mitochondrial disorders: clinical presentation and diagnostic dilemmas. Journal of inherited metabolic disease. 2003 Mar;26(2–3):199–207. doi: 10.1023/a:1024489218004 12889661

[5] Ng YS, Turnbull DM. Mitochondrial disease: genetics and management. Journal of neurology. 2016 Jan;263(1):179–91. doi: 10.1007/s00415-015-7884-3 26315846 PMC4723631

[6] Pal A, Banerjee S. Mitochondrial replacement therapy—a new remedy for defects in reproduction. Indian J Anim Sci. 2018 Jun 1;88(6):637–44.

[7] Picard M, Wallace DC, Burelle Y. The rise of mitochondria in medicine. Mitochondrion. 2016 Sep 1;30:105–16. doi: 10.1016/j.mito.2016.07.003 27423788 PMC5023480

[8] Smuts I, Louw R, Du Toit H, Klopper B, Mienie LJ, Van der Westhuizen FH. An overview of a cohort of South African patients with mitochondrial disorders. Journal of Inherited Metabolic Disease: Official Journal of the Society for the Study of Inborn Errors of Metabolism. 2010 Dec;33:95–104. doi: 10.1007/s10545-009-9031-8 20135231

[9] Karaa A, Elsharkawi I, Clapp MA, Balcells C. Effects of mitochondrial disease/dysfunction on pregnancy: a retrospective study. Mitochondrion. 2019 May 1;46:214–20. doi: 10.1016/j.mito.2018.06.007 29990538

[10] Afrifa J, Zhao T, Yu J. Circulating mitochondria DNA, a non-invasive cancer diagnostic biomarker candidate. Mitochondrion. 2019 Jul 1;47:238–43. doi: 10.1016/j.mito.2018.12.003 30562607

[11] Breton CV, Song AY, Xiao J, Kim SJ, Mehta HH, Wan J, et al. Effects of air pollution on mitochondrial function, mitochondrial DNA methylation, and mitochondrial peptide expression. Mitochondrion. 2019 May 1;46:22–9. doi: 10.1016/j.mito.2019.04.001 30980914 PMC6506186

[12] Pulkes T, Hanna MG. Human mitochondrial DNA diseases. Advanced drug delivery reviews. 2001 Jul 2;49(1–2):27–43. doi: 10.1016/s0169-409x(01)00124-7 11377801

[13] Forbes-Hernández TY, Giampieri F, Gasparrini M, Mazzoni L, Quiles JL, Alvarez-Suarez JM, et al. The effects of bioactive compounds from plant foods on mitochondrial function: A focus on apoptotic mechanisms. Food and Chemical Toxicology. 2014 Jun 1;68:154–82. doi: 10.1016/j.fct.2014.03.017 24680691

[14] Houston BA, Judge DP, Brown E, Halushka M, Barouch LA. Giant Ring Mitochondria in a Patient With Heart Failure and Cerebral White Matter Disease Resulting From an MT-TL1 Mitochondrial Gene Mutation. Journal of Cardiac Failure. 2017 Aug 1;23(8):652–5. doi: 10.1016/j.cardfail.2017.06.001 28624653

[15] Karaa A, Goldstein A, Balcells C, Mann K, Stanley L, Yeske PE, et al. Harmonizing care for rare diseases: how we developed the mitochondrial care network in the United States. Molecular Genetics and Metabolism. 2019 Jun 1;127(2):122–7. doi: 10.1016/j.ymgme.2019.05.012 31138493

[16] DiMauro S, Schon EA. Mitochondrial respiratory-chain diseases. New England Journal of Medicine. 2003 Jun 26;348(26):2656–68. doi: 10.1056/NEJMra022567 12826641

[17] Reinecke CJ, Koekemoer G, Van der Westhuizen FH, Louw R, Lindeque JZ, Mienie LJ, et al. Metabolomics of urinary organic acids in respiratory chain deficiencies in children. Metabolomics. 2012 Apr;8(2):264–83.

[18] Committee TM, Haas RH, Parikh S, Falk MJ, Saneto RP, Wolf NI, et al. The in-depth evaluation of suspected mitochondrial disease. Molecular genetics and metabolism. 2008 May 1;94(1):16–37. doi: 10.1016/j.ymgme.2007.11.018 18243024 PMC2810849

[19] Menezes MJ, Riley LG, Christodoulou J. Mitochondrial respiratory chain disorders in childhood: insights into diagnosis and management in the new era of genomic medicine. Biochimica et Biophysica Acta (BBA)-General Subjects. 2014 Apr 1;1840(4):1368–79. doi: 10.1016/j.bbagen.2013.12.025 24380876

[20] Taivassalo T, Ayyad K, Haller RG. Increased capillaries in mitochondrial myopathy: implications for the regulation of oxygen delivery. Brain. 2012 Jan 1;135(1):53–61. doi: 10.1093/brain/awr293 22232594 PMC3267980

[21] Bernier FP, Boneh A, Dennett X, Chow CW, Cleary MA, Thorburn DR. Diagnostic criteria for respiratory chain disorders in adults and children. Neurology. 2002 Nov 12;59(9):1406–11. doi: 10.1212/01.wnl.0000033795.17156.00 12427892

[22] Koene S, Hendriks JC, Dirks I, De Boer L, De Vries MC, Janssen MC, et al. International paediatric mitochondrial disease scale. Journal of Inherited Metabolic Disease: Official Journal of the Society for the Study of Inborn Errors of Metabolism. 2016 Sep;39(5):705–12. doi: 10.1007/s10545-016-9948-7 27277220 PMC4987390

[23] Parikh S, Goldstein A, Koenig MK, Scaglia F, Enns GM, Saneto R, et al. Diagnosis and management of mitochondrial disease: a consensus statement from the Mitochondrial Medicine Society. Genetics in Medicine. 2015 Sep;17(9):689–701. doi: 10.1038/gim.2014.177 25503498 PMC5000852

[24] Phoenix C, Schaefer AM, Elson JL, Morava E, Bugiani M, Uziel G, et al. A scale to monitor progression and treatment of mitochondrial disease in children. Neuromuscular Disorders. 2006 Dec 1;16(12):814–20. doi: 10.1016/j.nmd.2006.08.006 17123819

[25] Wolf NI, Smeitink JA. Mitochondrial disorders: a proposal for consensus diagnostic criteria in infants and children. Neurology. 2002 Nov 12;59(9):1402–5. doi: 10.1212/01.wnl.0000031795.91814.d8 12427891

[26] Hettiarachchi D, Lakmal K, Dissanayake VH. Mitochondrial diseases in South Asia–A systematic review. Mitochondrion. 2022 Jan 1;62:24–30. doi: 10.1016/j.mito.2021.10.007 34740865

[27] Meldau S, De Lacy RJ, Riordan GT, Goddard EA, Pillay K, Fieggen KJ, et al. Identification of a single MPV17 nonsense‐associated altered splice variant in 24 South African infants with mitochondrial neurohepatopathy. Clinical genetics. 2018 May;93(5):1093–6. doi: 10.1111/cge.13208 29318572

[28] Van Der Walt EM, Smuts I, Taylor RW, Elson JL, Turnbull DM, Louw R, et al. Characterization of mtDNA variation in a cohort of South African paediatric patients with mitochondrial disease. European Journal of Human Genetics. 2012 Jun;20(6):650–6. doi: 10.1038/ejhg.2011.262 22258525 PMC3355259

[29] Miller RL, Brewer JD, editors. The AZ of social research: A dictionary of key social science research concepts. Sage; 2003 Mar 21.

[30] Bajgain KT, Badal S, Bajgain BB, Santana MJ. Prevalence of comorbidities among individuals with COVID-19: A rapid review of current literature. American journal of infection control. 2021 Feb 1;49(2):238–46. doi: 10.1016/j.ajic.2020.06.213 32659414 PMC7351042

[31] Saleh J, Peyssonnaux C, Singh KK, Edeas M. Mitochondria and microbiota dysfunction in COVID-19 pathogenesis. Mitochondrion. 2020 Sep 1;54:1–7. doi: 10.1016/j.mito.2020.06.008 32574708 PMC7837003

[32] Grier J, Hirano M, Karaa A, Shepard E, Thompson JL. Diagnostic odyssey of patients with mitochondrial disease: Results of a survey. Neurology Genetics. 2018 Apr 1;4(2). doi: 10.1212/NXG.0000000000000230 29600276 PMC5873725

[33] Davison J, Lemonde H, Rahman S. Inherited mitochondrial disease. Paediatrics and Child Health. 2019 Mar 1;29(3):116–22.

[34] Esterhuizen K, Van der Westhuizen FH, Louw R. Metabolomics of mitochondrial disease. Mitochondrion. 2017 Jul 1;35:97–110. doi: 10.1016/j.mito.2017.05.012 28576558

